# A novel small molecule that selectively inhibits glioblastoma cells expressing EGFRvIII

**DOI:** 10.1186/1476-4598-6-30

**Published:** 2007-04-16

**Authors:** Dimitri G Trembath, Anita Lal, David J Kroll, Nicholas H Oberlies, Gregory J Riggins

**Affiliations:** 1 Department of Neurosurgery, Johns Hopkins University School of Medicine, Baltimore, Maryland 21231, USA; 2 Brain Tumor Research Center, Department of Neurological Surgery, University of California San Francisco, CA 94143, USA; 3 Natural Products Laboratory, Research Triangle Institute, Research Triangle Park, NC 27709, USA

## Abstract

**Background:**

Mutations of the epidermal growth factor receptor (EGFR) are a possible molecular target for cancer therapy. EGFR is frequently amplified in glioblastomas and 30 to 40% of glioblastomas also express the deletion mutation EGFRvIII. This frequent oncogenic mutation provides an opportunity for identifying new anti-glioblastoma therapies. In this study, we sought small molecule inhibitors specific for cancer cells expressing EGFRvIII, using isogenic parental cells without EGFRvIII as a control.

**Results:**

A screen of the NCI small molecule diversity set identified one compound, NSC-154829, which consistently inhibited growth of different human glioblastoma cells expressing EGFRvIII, but permitted normal growth of matched control cells. NSC-154829 had no previously established medicinal use, but has a purine-like structural component. Further experiments showed this compound increased apoptosis in cells with EGFRvIII, and moderately affected the expression of p21, independent of any changes in p53 levels or in Akt phosphorylation.

**Conclusion:**

These initial results suggest that NSC-154829 or a closely related structure might be further investigated for its potential as an anti-glioblastoma drug, although its precise molecular mechanism is still undefined.

## Background

The epidermal growth factor receptor (EGFR), a type I tyrosine kinase receptor, has been associated with numerous malignancies including breast, lung, head and neck, bladder, colorectal, ovarian, and prostate carcinomas, as well as with the most common form of brain tumor, glioblastoma [1]. Several small molecules have been developed to target EGFR including gefitinib (Iressa) and erlotinib (Tarceva), which interfere with ATP-binding and tyrosine kinase activity. EGFR inhibitors have shown promise and extended patient survival in lung, pancreatic and other cancers, however, survival gains are often modest, and, in non-small-cell lung cancers, activity is limited to the approximately 10% of patients with small activating mutations in the EGFR tyrosine kinase domain [2,3]. It also appears that subsequent mutations at different amino acids, also in the kinase domain, can confer drug resistance [4].

Targeting EGFR in glioblastomas has the additional challenge of the expression of EGFRvIII (epidermal growth factor receptor variant type III; also named de2-7 EGFR and deltaEGFR). EGFRvIII is found in 67% of tumors with amplified EGFR [5] and reported in 38% of all glioblastomas [6]. There has been recent evidence that EGFRvIII is also present in a minority (5%) of squamous cell lung cancers [7]. EGFRvIII is a deletion between exons 2–7 of the *EGFR *gene with loss of 267 amino acids from the extracellular domain, creating a constitutively active version of the protein [8]. EGFRvIII exists at high frequency in glioblastomas, and according to some reports imparts a worse prognosis and confers therapeutic resistance [9-13]. Our earlier work demonstrated that EGFRvIII expression in glioblastoma cells increased cellular motility and *in vitro *invasiveness [14]. In terms of current EGFR therapies, the picture in regards to glioblastomas is mixed. The kinase domain mutations correlated with gefitinib response are infrequent in glioblastomas and phase II trials of gefitinib showed no survival benefit in glioblastoma [15,16]. Yet, in a more recent study, tumors with both EGFRvIII and PTEN mutations responded better to EGFR inhibitors erlotinib or gefitinib [17]. However, since the present EGFR inhibitors have, at best, a small survival benefit in glioblastomas and as their use may select for further resistance-conferring mutations, there is utility in identifying additional compounds that can specifically inhibit cells with the EGFRvIII mutation.

**Figure 1:**
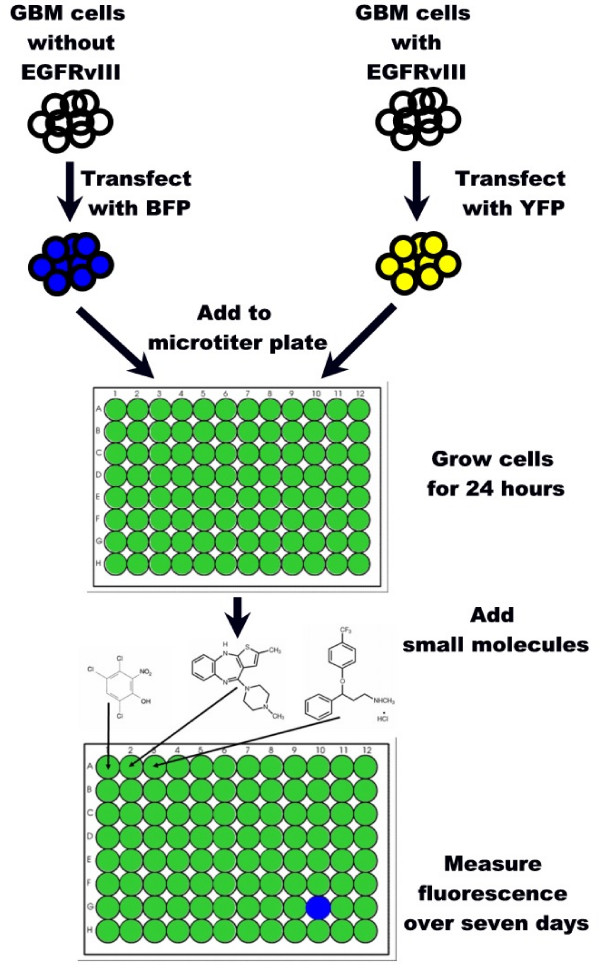
Diagram demonstrating the approach to screening small molecule libraries using cell lines that a) differ by a mutation of interest and b) are transfected with different fluorescent proteins. Cells with wild-type EGFR are transfected with blue fluorescent protein while their mutant counterparts, expressing EGFRvIII, are transfected with yellow fluorescent protein. Equal numbers of the transfected cells are co-cultured in the individual wells of a microtiter plate and, if left untreated, produce combined blue and yellow fluorescence. Appropriate concentrations of small molecules (1 small molecule per well) are then added and fluorescence levels measured over seven days. Well G10 would be considered a "hit" as the small molecule is reducing the growth of the mutant cell line, leaving only a blue fluorescent signal.

To find new inhibitors of glioblastoma cells expressing EGFRvIII, we used an isogenic cell-based approach for screening small molecule libraries [18]. For this study we stably transfected an established glioblastoma cell line with EGFRvIII using antibiotic selection. Generally, glioblastoma cell lines lose their native EGFRvIII over time when passaged *in vitro*, making it necessary to replace this oncogene to study it *in vitro*. The two isogenic cell lines (with and without EGFRvIII) were transfected with yellow or blue fluorescent protein respectively, and then these different fluorescent markers were used to independently track the growth of the two cell lines. Individual diverse small molecules were dissolved in the media of different multititer plate wells, each containing an identical co-culture of the mutant and control cells. *In vitro *growth of each cell line, and its response to the different small molecules, was monitored by measuring fluorescence levels over one week. In this manner, compounds that specifically inhibit the growth of the mutant-containing cell line were identified (see Figure 1). We applied this isogenic cell line screening strategy to the National Cancer Institute's diversity set of 1,990 small molecules to identify growth inhibitors of EGFRvIII-containing cells.

## Results

### Identification of NSC-154829

**Figure 2:**
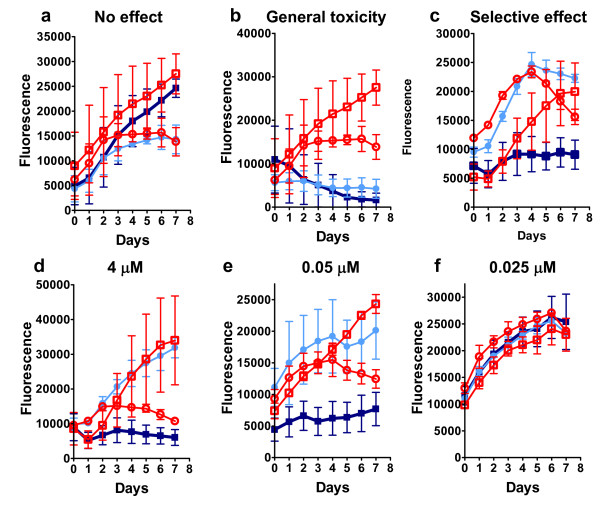
Examples of EGFRvIII and control cell growth responses to small molecule diversity set compounds. a) Most compounds showed no effect on either cell line. The two matched cell lines with no drugs (red circle = 897 and red square = 898) have a growth curve similar to the matched lines grown with a drug (light blue circle = 897, dark blue square = 898). Under normal circumstances, the 898 cells with EGFRvIII (square symbols), will eventually overtake the 897 mutant free cells (circle symbols) within the wells of the 96 well plate. b) Those compounds with a general toxic effect are apparent because both 897 and 898 will stop growing in response to drug treatment compared to cells without drug. c) Single inhibition of the 898 mutant cells as shown indicates a possible hit on the primary screen. d-f: Example of secondary screen results showing concentration effect on EGFRvIII containing cells. d) 4 μM concentration. e) 0.05 μM concentration f) 0.025 μM concentration. Each point represents an average of four experiments. Growth curves for mutant containing cells (898) are labeled as follows: dark blue square is with compound; red square is without compound. Isogenic cells without EGFRvIII (897) are labeled as follows: light blue circle is with compound; red circle is without compound.

Our primary small molecule screen identified 118 compounds with one of three properties: 1) inhibition of both cell lines, 2) preferential inhibition of one cell line, or 3) stimulation of both cell lines. Figure 2a–c shows examples of non-selective and selective inhibition (b and c respectively) contrasted with an example of non-inhibition (a). Of the 1,990 compounds, 118 (6%) had an observable effect on cell growth at 2 μM, a result similar to the 5.7% reported for the screen of *K-Ras *inhibitors [18]. These 118 compounds were re-screened at concentrations ranging from 0.03 to 4 μM to determine if there was a dose-dependent effect on the EGFRvIII-containing cell line (Figure 2d–f). In this secondary screen, 36 compounds inhibited both cell lines across different concentrations, and of these, 15 demonstrated a preferential inhibition of the EGFRvIII cell line in the primary screen. Accordingly, these 15 inhibitory compounds were submitted to a tertiary screen, but now against two different subclones of the 897 (control) and 898 (EGFRvIII) cell lines. Seven compounds that did not inhibit all of the 898 subclones were eliminated. The remaining eight compounds consistently and preferentially inhibited growth of the 898 cell lines and did not inhibit growth of the 897 cell lines.

### Verification of NSC-154829's selectivity against EGFRvIII + cells

**Figure 3:**
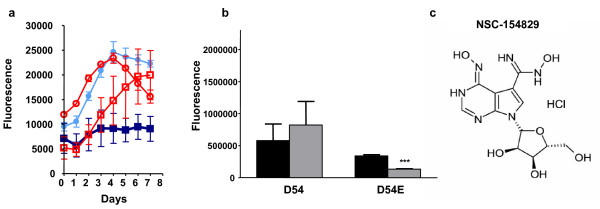
a) Initial growth response curve for 897 and 898 treated with NSC-154829. Growth curves for mutant containing cells (898) are labeled as follows: dark blue square, with compound; red square, without compound. Isogenic cells without EGFRvIII (897) are labeled as follows: light blue circle, with compound; red circle, without compound. Each point represents an average of duplicate experiments. b) Initial screens at 2 μM showing the selective effect of NSC-154829 on D54E cells, demonstrating efficacy across different genetic backgrounds. *** p < 0.0001. Further screening (see Table 1) would demonstrate significantly different IC_50_s for the D54 and D54E cell lines when treated with NSC-154829. c) Structure of NSC-154829, showing purine backbone.

To insure that the selectivity against cells expressing EGFRvIII was not a function of genetic background, two additional tests were performed on the remaining eight compounds: 1) the compounds were tested against additional 897 and 898 subclones (again transfected with blue and yellow fluorescent protein respectively), to determine if there were consistent significant difference in IC_50_s between the wild type and mutant cells and 2) the compounds were tested against a different glioma cell line, D54 and D54E, the latter transfected with EGFRvIII. Testing using different 897/898 subclones was performed using the same fluorescent screen as described earlier. IC_50 _values for one of the compounds, NSC-154829, are shown in Table 1; for each set of subclones, there was a significant lower IC_50 _for the 898 cells compared to the 897s. For testing the compounds against the D54 and 54E cell lines, cells were plated in individual wells and treated with the eight compounds that demonstrated preferential activity against the 898 cells. Growth was measured using a SybrGreen fluorescence assay [19]. Using this approach, NSC-154829 was the sole compound out of the original 1,990 that consistently showed preferential inhibition of EGFRvIII + cell lines in both the 897/898 and D54/54E systems. Figure 3 shows the preferential inhibition of one of the 898 subclones (a), the significant decrease in cell growth for the D54E cell line at 2 μM (b), and the structure of NSC-154829 (c). D54E also demonstrated a significantly lower IC_50 _when treated with NSC-154829, compared to D54 (see Table 1).

**Table 1:** IC_50_data for NSC-154829

*NSC Number*	*IC*_50_*897/898 clone 1*	*IC*_50_*897/898 clone 2*	*IC*_50_*897/898 clone 3*	*IC*_50 _*D54/54E trial #1*	*IC*_50 _*D54/54E trial #2*	*IC*_50 _*D54/54E trial #3*
154829	0.77 (0.71–0.84); 0.56 (0.51–0.69);	0.63 (0.49–0.81); 0.36 (0.35–0.38)	0.62 (0.54–0.71); 0.27 (0.24–0.30);	0.52 (0.43–0.63); 0.36 (0.27–0.48)	0.66 (0.52–0.84); 0.32 (0.21–0.5);	0.53 (0.42–0.68); 0.40 (0.34–0.46)
	**p = 0.003**	**p = 0.0009**	**p < 0.0001**	**p = 0.02**	**p = 0.004**	**p = 0.03**

p values represent IC_50 _comparisons between wild type and EGFRvIII + cells. Initial numbers represent the mean IC_50 _and 95% confidence intervals for wild type cells (either 897 or D54), the latter the mean IC_50 _and 95% confidence intervals for EGFRvIII + cells (either 898 or D54E). Each individual experiment was performed in sextuplicate

### Relative purity of NSC-154829

The relative purity of small molecules from the NCI library with selective activity against 898 cells was assessed by HPLC, since degradation is a potential problem in chemical libraries. NSC-154829 demonstrated 82% purity, using an area-under-the-curve analysis.

### NSC-154829's effects on cell growth

**Figure 4:**
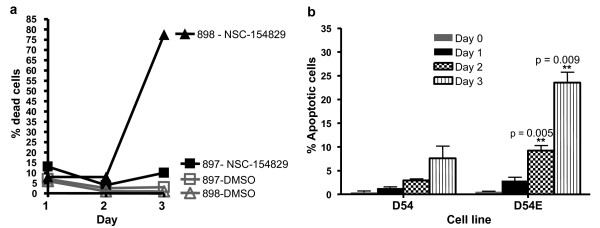
a) Trypan blue studies of cell viability following treatment with NSC-154829. Wild-type (897) or mutant (898) cells were plated and treated for either 24, 48, or 72 hours with 2 μM of NSC-154829. Cell viabilities remain similar until 72 hours when cell death increases dramatically in the 898 cell line compared to similarly treated 897 cells and DMSO-only treated controls. b) Hoechst studies for apoptosis following treatment with NSC-154829. D54 and D54E cells were treated with 2 μM NSC-154829 and then trypsinized, collected and stained with Hoechst 33258 at days 0, 1, 2 and 3. EGFRvIII+ cells showed a consistent and significant increase in the number of apoptotic nuclei compared to wild type.

Trypan blue studies were performed on 897 and 898 cells treated with 2 μM of NSC-154829. The percentage of dead cells remained similar between treated cell lines versus controls during the first 24–48 hours following treatment; however, at 48 hours, the percentage of dead cells present in the EGFRvIII+ cell line increased dramatically and significantly over wild-type (see Figure 4a). Hoechst staining of treated cells, examined on days 0–3, showed a significant increase in the number of apoptotic nuclei in the EGFRvIII + cells compared to controls (see Figure 4b).

### NSC-154829's effects on apoptosis

**Figure 5:**
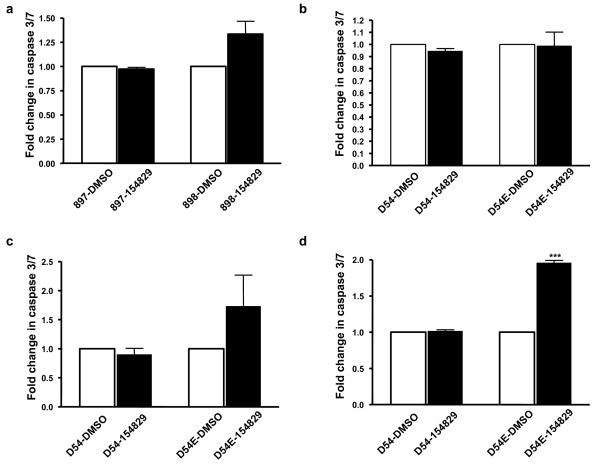
a) Effects on caspase 3 and 7 expression in 897 and 878 cells by treatment with 1 μM NSC-154829. Cells were exposed to 1 μM NSC-154829 and then caspase 3 and 7 levels measured as outlined in the Materials and Methods. Caspase levels were normalized to DMSO-only treated controls and expressed as fold increases. Each experiment was performed three times and each time in sextuplicate. There was borderline significance between the increase in caspase 3 and 7 levels in the mutant cells compared to wild type (p = 0.05). b) Effects of 1 μM NSC-154829 on caspase 3 and 7 levels in the D54/54E cell lines. While there was an increase in caspase activity in the D54E cells in response to NSC-154829 treatment compared to D54s, this difference was not significant. Each experiment was performed three times in sextuplicate. c) Dose-response effect of NSC-154829 on levels of caspase 3 and 7 in the D54 and D54E cell lines. Treatment with 5 μM of NSC-154829 nearly doubled levels of caspase 3 and 7 in both the wild type (D54) and mutant (D54E) cells. d) Treatment with 10 μM of NSC-154829 doubled levels of caspase 3 and 7 in the mutant cells, but had little effect on the wild-type cell line (*** p < 0.0001). Caspase levels for each cell line were normalized to DMSO-only treated controls and expressed as fold-change. Each assay was performed three times in sextuplicate.

To verify that the cell death measured in our fluorescence and trypan blue studies were due to apoptosis, we investigated if treatment with NSC-154829 resulted in an increase in caspases, important mediators of apoptosis in mammalian cells [20,21]. As shown in Figure 5a, treatment of 898 subclones with as little as 1 μM of NSC-154829 increased caspase 3/7 activity 1.5 fold over treatment with the carrier molecule (DMSO) alone. Similar treatments of the EFGR wild type cell line (897) produced no detectable difference in caspase levels compared to controls (Figure 5a). These changes were seen across different 897/898 subclones (data not shown). To insure that the presence of the fluorescent proteins was not interfering with the caspase assay, similar experiments were performed with D54 and D54E. Treatment with 1 μM NSC-154829 produced minimally increased caspase levels in D54E, compared to those in the wild-type cell line (Figure 5b). However, 5 μM and 10 μM of NSC-154829 produced corresponding increases in caspase activity in the EGFRvIII+ cells and minimal, if any, changes in caspase levels in the wild-type cell line (see Figure 5c and 5d). Thus, the selective inhibition of cell growth in the EGFRvIII+ cell lines would appear to be due, in part, to increasing levels of apoptosis in the mutant cell lines.

### NSC-154829's effects on phosphorylated Akt (Ser473)

Studies of glioblastomas and EGFR have focused on the effects EGFRvIII has in up-regulating the PI3K/Akt pathway as the mechanism behind the tumorigenicity of EGFRvIII [22,23]. Inhibition of Akt phosphorylation, whether by Akt kinase inhibitors or anti-sense oligonucleotides, has shown promise in limiting tumor growth, including glioblastomas [24,25]. Thus, we analyzed NSC-154829's effects the phosphorylation of the serine 473 residue of Akt in our cell lines via Western blot. The 898 cell line shows mildly increased P-Akt levels in normal conditions (10% serum) and the absence of drug. Treatment with 2 μM of NSC-154829 produced minimal changes in P-Akt levels in both the wild type and mutant cells (data not shown).

**Figure 6:**
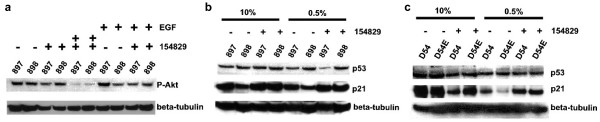
Western blot showing effect of 2 μM NSC-154829 on expression of phosphorylated Akt (serine 473; P-Akt) in both 897 and 898 cell lines (a), or on expression of p53 and p21 in the 897/898 and D54/54E cell lines under normal conditions (10% serum) or serum starvation (0.5% serum). a) Lanes 5–6 demonstrate the effect of serum starvation on the expression of P-Akt in both cell lines; this increased expression is diminished by treatment with 2 μM NSC-154829 (lanes 7 and 8). b) 897 and 898 cells were grown under conditions of either 10% serum (lanes 1–4) or 0.5% serum (lanes 5–8). Under conditions of 10% serum, exposure to 2 μM NSC-154829 has minimal effect on expression of p53, except for a mild decrease in the wild-type cells (897) under serum starvation conditions (lane 7). p21 expression increases for both the wild-type and mutant cells under both normal and serum starvation conditions; although the increase appears greater for the mutant cells (compare lanes 2 and 4). c) Effects of NSC-154829 on p53 and p21 in the D54 and 54E cell lines. Under conditions of 10% serum, there is minimal effect on p53 and a mild decrease in p21 signaling by NSC-154829. Serum starvation (0.5% serum) combined with NSC-154829 treatment produces an increase in p21 in both cell lines, mutant greater than wild-type (compare lanes 6 and 8), but does not appear to affect p53 expression.

It has been previously demonstrated that EGFRvIII is necessary for glioma cell proliferation under serum starvation conditions and that this proliferation is dependent on PI3K/P-Akt signaling [23,26]. When we subjected our 897/898 cell lines to serum starvation (0.5% serum), P-Akt levels were up-regulated in both the wild-type and mutant cell lines (see Figure 6a, lanes 1 and 2). When treated with NSC-154829, there is a dose-response effect, with increasing amounts of NSC-154829 (2 μM in Figure 6a, lanes 3 and 4; 5 μM Figure lanes 5 and 6) leading to a corresponding decrease in expression of P-Akt. When 897 or 898 cells are exposed to EGF (final concentration 100 ng/mL) there is, not surprisingly, increased expression of P-Akt (Figure 6a, lanes 7 and 8) in both cell lines. Similarly, this increased expression is mildly abrogated by 2 μM of NSC-154829 in both the wild type and mutant cells (Figure 6a, lanes 9 and 10).

NSC-154829's effects on P-Akt expression in the glioma cell line D54/54E was more pronounced, but similar in both cell lines. There was minimal effect on P-Akt expression under conditions of 10% serum (data not shown). When the D54/54E cells were stressed, via exposure to 0.5% serum, P-Akt levels increased dramatically and the increase in P-Akt in both cell lines was decreased by exposure to 2 μM NSC-154829 (data not shown). This effect, however, was not consistent, with the decrease in P-Akt often greater in the wild-type cell line versus mutant as levels of NSC-154829 increased from 2 to 5 μM. Application of EGF (100 ng/mL) did not produce as profound an increase in P-Akt compared to the 897/898 cell lines and, again, application of NSC-154829 did not consistently decrease P-Akt expression in the mutant compared to the wild-type cell line. Given these results, it appears that NSC-154829's selective effect on the EGFRvIII+ cells is not mediated through P-Akt or intermediary signaling molecules between the receptor and P-Akt.

### NSC-154829's effects on the p53/p21 pathway

As it did not appear that NSC-154829 was acting selectively through the P-Akt pathway, we next studied the p53 pathway which is also frequently mutated in glioblastomas, particularly in "secondary" glioblastomas, which develop from low grade astrocytomas (WHO grade II) to anaplastic astrocytomas (WHO grade III) and finally to glioblastomas (WHO grade IV) [27]. The increased caspase activity seen in the EGFRvIII+ cells also suggested this pathway, given p53's role in apoptosis regulation.

For both cell lines there was little or no effect on p53 in response to 2 μM of NSC-154829, regardless of serum conditions (Figures 6b and 6c). The response of p21 was more variable. Figure 6b shows NSC-154829's effect on p21 in the 897/898 background. In the presence of 10% serum, there was a minor increase in p21 in the EGFRvIII+ cells when exposed to 2 μM of NSC-154829 (Figure 6b, lanes 1–4). This small effect was still present when the cells were stressed under conditions of 0.5% serum (Figure 6b, lanes 6 and 8). For the wild-type cells, under conditions of 0.5% serum p21 was unchanged in response to NSC-154829 (Figure 6b, lanes 5 and 7).

In the D54/54E background, levels of p21 decreased in both cell lines in response to drug treatment under conditions of 10% serum (lanes 1–4, Figure 6c), but increased in both cell lines, particularly in the EGFRvIII+ cells, when grown in 0.5% media and then exposed to NSC-154829 (Figure 6c, lanes 5–8). Although p21 levels were either increased with drug treatment, or were less down-regulated in the mutant cells for the conditions studied, the effect was likely not large enough to account for the growth differences observed with NSC-154829 treatment.

## Discussion

Glioblastoma multiforme (GBM) is the most common primary brain tumor with approximately 12,000 new cases a year in the United States [28]. Despite extensive research, the prognosis for GBM is poor, with a median survival of 10 to 12 months [29]. EGFR mutations have been demonstrated with a high frequency in glioblastomas with the majority of these tumors demonstrating increased expression of EGFR and a subset showing expression of the common deletion mutation EGFRvIII [5,6]. The latter renders these tumors resistant to radiation and to many of the commonly used tyrosine kinase inhibitors [12,30,31]. The prevalence of the EGFRvIII mutation, however, could prove to be a useful target for glioblastomas and other tumors with this mutation.

In this study, we used the expression of EGFRvIII to find a small molecule, NSC-154829, from the National Cancer Institutes Diversity Set, which selectively inhibits glioblastoma cells with this mutation, compared to glioblastoma cells without the mutation. Using a fluorescence-based screen, we found that NSC-154829 inhibits the growth of EGFRvIII + cells (898s), with IC_50_s of .27 to .59 μM compared to .62 to .77 μM for wild-type cells (897s; see Table 1). The activity across different subclones indicates that the inhibition is not unique merely due to particular genetic changes in one subclone, but instead may be due to changes induced by the presence of EGFRvIII. This conclusion is also supported by the preferential activity against EGFRvIII + cells from a different glioblastoma cell line, D54/54E. Again, IC_50_s for the EGFRvIII + cells (D54E) were significantly lower than that of the wild-type cells (an average of 0.33 μM for D54E compared to 0.57 μM for D54; see Table 1).

Supporting our screening results were trypan blue studies that showed the mutant cell line experiencing a dramatic increase in cell death following treatment with NSC-154829 (Figure 4a). Hoechst staining and caspase 3/7 studies support that this increase is due to an increase in apoptosis in the mutant cells lines, an effect not seen in the wild-type cells (Figures 4b and 5).

Effects on downstream molecules in the EGFR pathway were not immediately apparent in our studies. Akt has received extensive attention in the literature as a possible target for glioblastoma therapy [24,32]. However our Western blot studies showed non-selective effects on Akt phosphorylation in both the wild-type and mutant cell lines (Figure 6a), which implies that NSC-154829's preference for inducing apoptosis in the EGFRvIII cells is not mediated through differences in Akt phosphorylation.

NSC-154829's structure is similar to that of other purine analogue anti-metabolites, including compounds currently used as chemotherapeutics, such as fludarabine and cladribine [33,34]. Indeed these two drugs showed similar selectivity on our isogenic EGFRvIII lines (data not shown). Previous studies have shown these nucleoside analogues work, at least in part, by increasing apoptosis via up-regulation of p53 and/or p21 [35,36]. Our studies of p53 and p21 show no effect on p53, but show that p21 responds differently in the mutant cells in response to treatment with NSC-154829. However, there is no evidence for a direct molecular mechanism of action for NSC-154829 on the p53/p21 pathway and p21's differential response varies with cell background and serum conditions. This inconsistent effect may be secondary to other as yet undefined genetic differences between the two glioma cell lines. Overall, our experiments do not clarify the mechanism of NSC-154829's selective effect against EGFRvIII + cells and should not be interpreted as advocating a direct interaction between NSC-154829 and p21.

It is of interest that reports on similar novel purine analogues show different effects on p53 and p21. Radhakrishnan and Gartel (2006) recently identified a novel transcriptional inhibitor, NSC-188491, which has structural similarities to NSC-154829. NSC-188491 increased p53 levels, but repressed p21, hdm2, and survivin [37]. Park et al. (2006), however, found a more typical relationship between p53 and p21 with MCS-C2, a novel cyclin-dependent kinase inhibitor with structural similarities to NSC-154829. Treating prostate cancer cells with MCS-C2 induced apoptosis and produced up-regulation of p53 and p21 [38]. Thus, it would appear that the effects of nucleoside analogues such as NSC-154829 are mediated perhaps in part through the p53/p21 pathway, but these can vary, based on structure and/or genetic background of the cell line involved. Studying NSC-154829's effect on DNA synthesis and comparing these effects with those of existing purine analogues would be logical next steps.

Literature on NSC-154829 is minimal, however the 3D Mind website indicates this small molecule maps to cluster k18.17, a cluster that does not have a specific activity, but includes agents such as anti-mitotics [39]. Other small molecules that map to this cluster have vague structural similarities; none, however, were included in the diversity set for testing. Of those small molecules that initially showed selective activity against the 898 cell line, but which did not selectively inhibit the D54E cells (data not shown), NSC-321239 and NSC-75140 both had similar purine-type backbones to that of NSC-154829. NSC-188491 (map location: cluster k18.13) was included in our diversity set and showed efficacy against EGFRvIII+ cells in our primary screen, but did not demonstrate a significant IC_50 _difference in the background of D54/54E (data not shown).

The presence of EGFRvIII induces many other transcript and protein changes and some of these may be the critical steps affected by NSC-154829 [14]. The advantage to the screen described above is that it targets a mutation frequently and almost exclusively found in GBMs, an approach previous glioma-centered small molecule screens have not exploited [32,40,41]. However, this work is preliminary and we acknowledge that this inhibitor is unlikely to act as a single therapy to overcome EGFR signaling in glioblastomas because 1) drug resistance is a frequent problem with single modality therapy, 2) the EGFR pathway can be activated at a point downstream of EGFR in glioblastoma, and 3) glioblastoma cell lines and xenografts thrive after loss of EGFRvIII. However, it is important to note that our approach does not find compounds that directly interact with EGFRvIII, but likely target a downstream gene product induced by EGFRvIII pathway activation, and could lead to a therapy synergistic with those that inhibit EGFR directly. For future work it would be useful to test NSC-154829 in combination with known EGFR inhibitors and/or proven glioblastoma therapies such as radiation. The latter may be important, since EGFRvIII is reported to also enhance radiation resistance [12]. In addition, analogues of NSC-154829 should be tested to determine if its therapeutic index and drug delivery properties can be improved.

## Conclusion

This study helps demonstrate the potential of cell-based screening to rapidly identify inhibitors of cells with a cancer-associated mutation. Although screens using isogenic cells with and without a mutation do not initially reveal the inhibitor's direct molecular mechanism, in our case it is likely that the target is related to EGFRvIII pathway activation. In theory, screens such as the one described above may identify compounds that are specific to a cancer-causing mutation, but at the risk of yielding a lower percentage of "hits" and ones with highly toxic side effects, compared to traditional cell based screens [18]. Given these limitations, determining *in vivo *efficacy and the maximum tolerated doses are the next steps in the investigation of NSC-154829. Our findings suggest that NSC-154829, or closely related structures, should be investigated further for treatment of cancers with EGFR pathway activation.

## Methods

### Cell lines and plasmids

Blue and yellow fluorescent protein (BFP and YFP, respectively) expression vectors were kind gifts from Bert Vogelstein and described previously [18]. The engineering of U87 and D54 glioblastoma cell lines either expressing (898, D54E) or not expressing EGFRvIII (897, D54) was also described previously [14]. Stable transfectants were created using Transfast™ (Promega, Madison, WI), and subclones were isolated by puromycin selection. Fluorescence measurements were compared between cell lines, and different clones were chosen based on the expression of equal levels of either fluorescent signal over time.

### Verification of EGFRvIII expression

Comparative EGFRvIII expression in D54E and D54 cells has previously been described [14]. RNA expression of EGFRvIII in 898s was verified using RT-PCR and primers flanking the deletion site. Western blots were performed on cell lysates of each individual 897 and 898 clone, as well as lysates from the D54 and D54E cell lines to verify EGFRvIII protein expression (data not shown).

### Screening procedure

The diversity set of 1,990 synthetic compounds, derived from a larger collection of almost 140,000 compounds, was obtained with permission from the Developmental Therapeutics Group of the National Cancer Institute [42]. A mixture of equal amounts of 10,000 cells each of 897 (wild-type EGFR) and 898 (EGFRvIII mutant) transfected with BFP or YFP, respectively, were cultured in 96 well plates (200 μL final volume per well) containing Dulbecco's Modified Eagle's Medium (DMEM) with 10% fetal calf serum and puromycin (500 ng/mL). Controls consisted of wells without cells to measure background fluorescence. Twenty-four hours later, 2 μM of each compound in DMSO was added individually to wells in columns 2–11; wells in column 1 received DMSO only. Fluorescence was measured before compound addition and subsequently every 24 hours for 7 days following compound addition, using a Genios system (Tecan Instruments, Research Triangle Park, North Carolina). Absorption/emission spectra for the two fluorescent proteins were 390 nm absorption/510 nm emission for BFP and 530 nm absorption/590 nm emission for YFP. This primary screen was performed in duplicate in independent experiments. Fluorescence data were exported into GraphPad Prism^®^, Version 4 (San Diego, CA) and growth curves were plotted. Secondary screens were performed on compounds that demonstrated selective inhibition, using concentrations ranging from 0.03 to 4 μM. Tertiary screens were performed with compounds at 2 μM concentration against two new additional subclones containing the blue or yellow fluorescent proteins. Both the secondary and tertiary screens were performed in four independent runs and used to calculate the mean and standard error of the mean for each daily fluorescence level.

For experiments with D54 and D54E, 3,000 cells from each cell line were plated in individual wells of a 96 well plate (Day 0). The next day (Day 1), the cells were treated with either DMSO (Hybrimax™, Sigma-Aldrich, St. Louis, MO) or with 2 μM of the small molecule of interest. On Day 4, the media was removed, the cells were lysed with 50 μL of 0.2%SDS, incubated at 37°C for two hours, then 150 μL of a 1:750 solution of SYBR Green I nucleic acid stain (Molecular Probes, Eugene, OR) added. Fluorescence was measured on a Perkin Elmer Wallac 1420 Multilabel counter (Perkin Elmer, Turku, Finland) with a 485 nm excitation filter and a 535 nm emission filter.

### Statistical analyses

Fluorescence levels were measured daily and results averaged for each day, including standard error of the mean. Growth curves were plotted and compared between each cell line with and without drug. Linear regression analysis was used to identify differences between 897 and 898 cell line growth curves in response to compound treatment, compared to the DMSO-only treated controls, using a p-value < 0.05 as the criteria for significance. IC_50 _values were determined by comparing fluorescence values for the 898 or 897 cell lines with drug to 898 or 897 without drug respectively, then transforming and normalizing the data using GraphPad Prism.

For experiments with D54 and D54E cells, fluorescence levels were measured on day 4 post drug addition and results averaged to give a mean and standard error of the mean. A non-paired T-test was performed to identify differences in fluorescent levels between DMSO-only and compound-treated cells with p-values < 0.05 considered significant. Outliers were excluded using Grubbs' test.

### Compound purity

The relative purities of six of the eight compounds identified as hits were assessed by HPLC (Waters 600E multisolvent delivery system, Milford, MA; HP Chemstation, Palo Alto, CA) as measured via an evaporative light-scattering detector (ELSD; S.E.D.E.R.E., Alfortville, France), as this technology does not depend on a UV-active chromophore. The other two compounds, NSC-73300 and NSC-609699, were not available in sufficient amounts for this test and omitted. All analyses of 30 min utilized an Inertsil ODS 3 column (5 μm, 4.6 × 250 mm; MetaChem Technologies, Torrence, CA). As each compound had slightly different chromatographic properties, individualized mobile phases of varying concentrations of HPLC-grade MeOH and H_2_O were developed, and the latter had either trifluoroacetic acid (TFA) or triethylamine (TEA) added as modifiers to enhance the chromatographic separations. Using an isocratic system 80:20 MeOH:H_2_O (0.1%TFA), NSC-177407 dissolved in MeOH had a R_t _of 14.2 min. Using an isocratic system of 20:80 MeOH:H_2_O (0.1%TFA), NSC-75140 dissolved in MeOH had a R_t _of 5.6 min. Using a gradient system of MeOH:H_2_O (0.1%TFA) that ramped from 20:80 to 80:20 over 30 min, NSC-154829 dissolved in H_2_O had a R_t _of 10.9 min, and using the same system, NSC-321239 dissolved in DMSO had a R_t _of 14.3 min. Using a gradient system of MeOH:H_2_O (0.1%TFA) that ramped from 20:80 to 80:20 over 20 min and was then held isocratic thereafter, NSC-163443 dissolved in DMSO had a R_t _of 23.7 min. Using an isocratic system of 40:60 MeOH:H_2_O (0.05% TEA), NSC-100880 had a R_t _of 5.0 min.

### Trypan blue studies

100,000 cells from each cell line were plated individually and allowed to grow for 24 hours. NSC-154829 was added at a final concentration of 2 μM and cells washed, trypsinized, and collected at appropriate time points (24, 48, or 72 hours). The collected cells were mixed with trypan blue (Sigma-Aldrich, St. Louis, MO) and cell counts performed. 300 cells were counted for each experiment and each experiment was performed in duplicate.

### Hoechst studies

100,000 cells from each cell line were plated individually and allowed to grow for the specified time periods. NSC-154829 was added at a final concentration of 2 μM and then cells were washed, trypsinized, and collected at appropriate time points. The cells were resuspended in 40 μL of Hank's Balanced Salt Solution (Invitrogen, Carlsbad, CA, U.S.A.) and then mixed with 360 μL of Hoechst solution (300 μl PBS, 20 μl 10% NP-40, 36 μl 37% formaldehyde and 4 μl 1 mg/ml Hoechst 33258 [Invitrogen, Carlsbad, CA, U.S.A.]). A minimum of 300 nuclei were then evaluated using fluorescent microscopy. Each time point was evaluated in triplicate and increases in number of apoptotic nuclei were evaluated using a student's T-test.

### Apoptosis assays

Apoptosis assays were performed using the Caspase-Glo^® ^3/7 Assay (Promega Corporation, Madison, WI, U.S.A.) according to manufacturer's instructions. Briefly, 5,000 cells were plated in 100 μL media and allowed to grow for 24 hours. NSC-154829 was then added at an appropriate final concentration (1, 2, 5, or 10 μM). Cells were treated for 24 hours, and then the plates removed from the incubator and allowed to equilibrate to room temperature. 100 μL of Caspase-Glo^® ^3/7 reagent was added to each well, the contents mixed, and then the plate incubated for 1 hour at room temperature. Luminescence was measured on a Perkin Elmer Wallac 1420 Multilabel counter (Perkin Elmer, Turku, Finland) per manufacturer instructions. Luminescence levels of NSC-154829 treated wells were normalized to DMSO-treated controls and fold increases in caspase 3/7 levels compared using student's T-test.

### Western blotting

40,000 cells were plated in individual wells of six-well tissue culture plates, allowed to grow overnight, and then treated with either DMSO or appropriate concentrations of NSC-154829 (2, 5 or 10 μM) for 24 or 96 hours. Media was removed, the cells were washed with 1x PBS, trypsinized, and collected. Cells were lysed and protein collected using Pierce NE-PER^® ^Nuclear and Cytoplasmic Extraction Reagents (Rockford, IL, U.S.A.), following manufacturer's instructions. Protein extracts were quantified using the Pierce BCA™ Protein Assay Kit. Equal amounts were loaded onto NuPage^® ^Novex 4–12% Bis-Tris gels (Invitrogen, Carlsbad, CA, U.S.A.) and electrophoresed for 1.5–2 hours at 80 volts. Proteins were transferred to Immunoblot PVDF membranes (Bio-Rad, Hercules, CA, U.S.A.), then probed with antibodies against phosphorylated Akt, EGFR, p53, p21 (Cell Signaling Technology, Danvers, MA, U.S.A), GAPDH (Santa Cruz Biotechnology, Inc., Santa Cruz, CA, U.S.A), or neuron specific beta-III tubulin (R&D Systems, Minneapolis, MN, U.S.A.). Appropriate horseradish peroxidase-conjugated secondary antibodies and the ECL Plus (GE Healthcare Bio-Sciences Corp., Piscataway, NJ, U.S.A.) were used for chemiluminescent detection. Following application of the chemiluminescent substrate, blots were exposed to Hyperfilm ECL (GE Healthcare Bio-Sciences Corp., Piscataway, NJ, U.S.A.) for appropriate periods of time.

For experiments involving serum starvation, appropriate numbers of cells were initially plated in media with 10% serum, allowed to grow for 24 hours, then the media removed; the cells were washed, and then maintained in media with 0.5% serum for the duration of the experiments. Cells stimulated with EGF were exposed to 100 ng/mL of EGF for 15 minutes and then cells were collected, lysed, and protein extracted as described above.

## Abbreviations

EGFR: epidermal growth factor receptor

EGFRvIII: epidermal growth factor receptor variant type III

TOP1: topoisomerase I

MeOH: methanol

## Competing interests

The author(s) declare that they have no competing interests.

## Authors' contributions

DGT drafted the manuscript, performed statistical studies, and carried out all experiments described in this paper, including: the creation of the fluorescently labeled 897 and 898 cell lines, drug screens, Western blots, apoptosis assays, RNA extractions, PCRs, trypan blue studies, and Hoechst studies. AL created the D54 and D54E cell lines. DJK and NHO performed studies of compound purity, participated in study design, and provided background and insight on hits from drug screens. GJR conceived of the study, participated in its design and helped draft and edit the manuscript. All authors read and approved the final manuscript.
